# Burnout among neurologists caring for patients with cognitive disorders in Spain

**DOI:** 10.1371/journal.pone.0286129

**Published:** 2023-05-25

**Authors:** Juan Fortea, Elena García-Arcelay, Guillermo Garcia-Ribas, Neus Canal, Jorge Maurino

**Affiliations:** 1 Department of Neurology, Hospital de la Santa Creu i Sant Pau, Barcelona, Spain; 2 Medical Department, Roche Farma, Madrid, Spain; 3 Department of Neurology, Hospital Universitario Ramón y Cajal, Madrid, Spain; 4 IQVIA, Barcelona, Spain; Houston Methodist Academic Institute, UNITED STATES

## Abstract

**Background:**

Physician burnout has a negative impact on both physicians and patients. Limited information is available on professional burnout of neurologists. The aim of this study was to assess the presence of burnout among neurologists caring for patients with cognitive disorders and to identify associated factors.

**Methods:**

An online, cross-sectional study was conducted in collaboration with the Spanish Society of Neurology. Neurologists involved in the care of patients with cognitive disorders answered a survey composed of demographic characteristics, professional background, clinical practice setting, and behavioral factors. Burnout was assessed using a single-item measure from the Physician Work Life Study. A multivariate logistic regression analysis was conducted to determine the association between neurologists’ characteristics and burnout.

**Results:**

A total of 188 neurologists answered the survey. The mean age (standard deviation-SD) was 40.6 (11.3) years and 52.7% were male. The majority of participants were general neurologists (60.6%) who attending a median of 20 patients with cognitive disorders (interquartile range 10.0–30.0) weekly. Thirty-nine participants (20.7%) reported burnout. Participants with burnout had greater experiences of regret associated with past clinical decisions than their counterparts (mean Regret Intensity Scale scores of 2.3 and 1.9, respectively; p = 0.003). Burnout was associated with non-academic practice (OR = 3.02 [95% CI 1.18, 7.73], p = 0.021) and care-related regret (OR = 2.53 [95% CI 1.13, 5.64], p = 0.023) in the multivariate analysis after adjustment for confounders.

**Conclusions:**

Professional burnout was a common phenomenon among neurologists managing cognitive disorders. Identifying physician burnout and its associated factors may be critical for implementing preventive intervention strategies.

## Introduction

Professional burnout is a syndrome resulting from chronic workplace stress that has not been successfully managed [1]. In recent years, there has been an increased interest in studying burnout among physicians, recognizing that this phenomenon is common and negatively influences their physical and emotional health and the overall quality of patient care [1–4].

Prevalence of burnout among physicians ranged from 7.7% to 43.2% in a recent systematic review of 56 studies in 41 European countries across multiple specialties [2]. Neurologists, emergency and general internal medicine physicians are three times more likely to suffer from burnout compared to other specialties [1]. A proportion of 60.1% of 1,671 neurologists surveyed by the American Academy of Neurology (AAN) reported at least one symptom of burnout [5]. More hours worked, more nights on-call, higher outpatient volume, and higher percentage of time in clinical practice were associated with higher burnout risk [6].

The management of patients with cognitive impairment and dementia involves a number of stress factors for neurologists, including disclosure of the diagnosis in a context of stigmatization of dementia in society, barriers to access to core Alzheimer´s disease biomarkers, and uncertainties related to new anti-amyloid-beta protein disease-modifying treatments [7–10]. However, information on burnout among neurologist managing cognitive disorders is limited. The aim of this study was therefore to assess the presence of burnout in neurologists caring for patients with cognitive disorders and to identify associated factors.

## Materials and methods

We conducted an online, non-interventional, cross-sectional study conducted in collaboration with the Spanish Society of Neurology (SEN). Neurologists involved in the management of patients with cognitive disorders were invited to participate in the study by e-mail. The study was approved by the Research Ethics Board of the Hospital Universitario Clínico San Carlos, Madrid, Spain. All participants provided a written informed consent.

Participants answered a survey assessing demographic characteristics, professional profile and clinical practice setting, attitudes toward adoption of evidence-based innovations, experiences of regret associated with past clinical decisions, and professional burnout. The Evidence-Based Practice Attitude Scale (EBPAS) is a 15-item instrument to assess healthcare professionals´ willingness to adopt new evidence-based treatments, interventions, and practices [11]. Total score ranges from 0 to 4, with higher scores indicating a more positive attitude toward innovations. The Regret Intensity Scale (RIS-10) is a 10-item validated tool to assess regret caused by a past event caring for a patient [12]. Each item is scored on a Likert scale ranged from 1 to 5. Higher scores indicate higher regret intensity. Burnout was assessed using a single-item measure from the Physician Work Life Study [13]. A cut-off score ≥3 indicates the presence of burnout.

The primary analysis assessed burnout and associated factors. A multivariate logistic regression analysis with backward selection was completed to determine the association between neurologists’ characteristics and burnout. All tests were 2-tailed, and p-values <0.05 were considered significant. We used STATA 17 (College Station, TX: StataCorp LP) to conduct all analyses.

## Results

Between 22 April to 28 June 2022, 1,580 neurologists were invited to participate in the study, 267 initiated the survey, and 188 completed all questionnaires (response rate of 11.9%). The mean age (standard deviation-SD) was 40.6 (11.3) years and 52.7% were male. Participants were predominantly general neurologists (60.6%) who attended a median of 20 patients with cognitive disorders (interquartile range 10.0–30.0) weekly. The main characteristics of the study population are shown in Table 1.

**Table 1:** Sociodemographic and professional characteristics of the study population.

	Burnout N = 39	No burnout N = 149	Total N = 188	p-value
Age, years, mean (SD)	41.8 (11.8)	40.3 (11.3)	40.6 (11.3)	0.484
Sex, male, n (%)	23 (59.0)	76 (51.0)	99 (52.7)	0.375
Specialization, n (%)				0.217
General neurology	27 (69.2)	87 (58.4)	114 (60.6)	
Cognitive disorders	6 (15.4)	33 (22.1)	39 (20.7)	
Practice setting, n (%)				0.106
Academic	28 (71.8)	124 (83.2)	152 (80.9)	
Non-academic	11 (28.2)	25 (16.8)	36 (19.1)	
Patients managed weekly,	16.0	20.0	20.0	0.425
median (IQR)	(15.0, 30.0)	(10.0, 30.0)	(10.0, 30.0)	
Number of outpatient visits	4.0	4.0	4.0	0.615
weekly, median (IQR)	(3.0, 5.0)	(3.0, 5.0)	(3.0, 5.0)	
Investigator in clinical trials, yes, n (%)	26 (66.7)	75 (50.3)	101 (53.7)	0.068
Authorship of peer-reviewed publications, yes, n (%)	30 (76.9%)	121 (81.2)	151 (80.3)	0.549
EBPAS score, mean (SD)	3.1 (0.5)	3.1 (0.5)	3.1 (0.5)	0.966
RIS-10 score, mean (SD)	2.3 (0.9)	1.9 (0.7)	2.0 (0.8)	**0.003**
RIS-10 score >3, n (%)	8 (20.5)	14 (9.4)	22 (11.7)	0.054

EBPAS = Evidence-Based Practice Attitude Scale; IQR = Interquartile range; RIS-10 = 10-item Regret Intensity Scale; SD = Standard deviation.

Thirty-nine participants (20.7%) reported burnout. Participants with burnout had higher levels of care-related regret than their counterparts (mean RIS-10 scores of 2.3 and 1.9, respectively; p = 0.003) (Table 1). “Emotions come back to me”, “I feel uncomfortable”, and “I feel devalued” were the participants´ feelings of regret with the greatest intensity. After adjustment for confounders, burnout was associated with non-academic practice (OR = 3.02 [95% CI 1.18, 7.73], p = 0.021) and care-related regret (OR = 2.53 [95% CI 1.13, 5.64], p = 0.023) in the multivariate analysis (Table 2).

**Table 2:** Univariate and multivariate logistic regression analysis.

Covariate	Class	Univariate			Multivariate*		
		OR	95% CI	p-value	OR	95% CI	p-value
Age		1.011	[0.98–1.04]	0.482	—	—	—
Sex	Man	Ref	—	—	—	—	—
	Woman	0.724	[0.36–1.48]	0.376	—	—	—
Number of visits weekly (*continuous*)		1.072	[0.82–1.41]	0.613	—	—	—
Number of visits weekly (*categorical*)	≤ 4	Ref	—	—	—	—	—
	> 4	0.927	[0.45–1.91]	0.837	—	—	—
Number of patients managed weekly *(continuous)*		0.992	[0.97–1.01]	0.424	—	—	—
Number of patients managed weekly *(categorical)*	≤ 20	Ref	—	—	—	—	—
	>20	0.786	[0.38–1.63]	0.518	—	—	—
Practice setting	Academic	Ref	—	—	Ref	—	—
	Non-academic	1.949	[0.86–4.42]	**0.110**	3.02	[1.18–7.73]	**0.021**
Specialization	No (general neurology)	Ref	—	—	—	—	—
	Yes	0.624	[0.29–1.33]	0.219	—	—	—
Specialization in cognitive disorders	No	Ref	—	—	—	—	—
	Yes	0.639	[0.25–1.66]	0.356	—	—	—
Investigator in CTs	No	Ref	—	—	Ref	—	—
	Yes	1.973	[0.94–4.13]	**0.071**	2.83	[0.84–9.52]	0.092
Number of CTs in the last 3 years *(continuous)*		1.050	[0.95–1.16]	0.320	—	—	—
Number of CTs in the last 3 years *(categorical)*	0	Ref	—	—	Ref	—	—
	≥1	1.699	[0.84–3.45]	**0.143**	1.78	[0.53–5.95]	0.347
Authorship of peer-reviewed publications	No	Ref	—	—	—	—	—
	Yes	0.771	[0.33–1.81]	0.549	—	—	—
RIS-10 score *(continuous)*		1.068	[1.02–1.12]	**0.004**	—	—	—
RIS-10 score (*categorical)*	>3	2.125	[1.03–4.38]	**0.041**	2.53	[1.13–5.64]	**0.023**
	≤3	Ref	—	—	Ref	—	—
EBPAS score		0.984	[0.47–2.07]	0.966	—	—	—

*Variables with p<0.2 in the univariate analysis were included.

CI = Confidence interval; CTs = Clinical trials; EBPAS = Evidence-Based Practice Attitude Scale; IQR = Interquartile range; OR = Odds ratio; Ref = Reference; RIS-10 = 10-item Regret Intensity Scale; SD = Standard deviation.

## Discussion

Burnout is a major international problem for healthcare services [1, 14, 15]. Neurology was one of the medical specialties with the highest reported burnout and lowest life-work integration satisfaction in a recent survey among 7,510 physicians in the US [16]. These problems seem to be even more evident among female neurologists [6].

A proportion of 57.4% of headache specialists suffer from burnout, whereas epilepsy specialists have a lower risk of burnout compared to those practicing general neurology [5, 17]. However, there is still limited information available on this phenomenon among the different subspecialties of neurology.

In our study, one in five neurologists caring for patients with cognitive disorders reported burnout. Non-academic practice and the experience of care-related regret were the associated factors in a population of mostly general neurologists with research activity.

In a survey conducted by the AAN, academic practice neurologists had a lower burnout rate and higher rates of career satisfaction and quality of life than non-academic neurologists [5]. These academy-setting neurologists worked more hours per week on average, spent a smaller proportion of their time on direct patient care and more time on research and teaching, while neurologists working in the non-academic setting spent more nights per week on-call, saw more patients per week and spent more weekends per year in the hospital [5]. Diversification into an academic practice including teaching and research may have some protective effect on burnout [18].

Regret is a negative emotion experienced when one believes that a situation would have had a better outcome if a different decision had been made [12, 19]. Although it is still an understudied phenomenon among healthcare professionals, care-related regret has been associated with sleep disturbances, poor quality of life, high job turnover, low commitment, absenteeism, and therapeutic inertia [19]. Intensity of past regret experiences and maladaptive coping strategies among physicians and nurses were associated with poor self-rated health, lower job satisfaction, and more sick leave [19].

There were no previous reports of burnout among neurologists attending cognitive disorders. Our results may have practical implications for the healthcare system in developing strategies to reduce burnout. The increasing number of patients with neurodegenerative disorders including Alzheimer’s disease due to ageing and the progressive shortage of neurologists make it crucial for policymakers and healthcare authorities to address the phenomenon of burnout to ensure the viability of the health system and adequate patient care [5, 8, 20]. Innovative strategies aimed at enabling neurologists to be more academically active and fostering their psychological resources to improve their ability to cope with negative experiences such as regret could contribute to mitigating different work stressors [14, 18].

Our study has some limitations. First, self-selection bias may have meant that neurologists who are more motivated or more knowledgeable about cognitive impairment and dementias may have completed the survey, impacting on the prevalence of burnout. Second, there could be a risk of nonresponse bias from neurologists who received the SEN invitation as a consequence of workload and burnout. Third, the cross-sectional study design may limit the ability to establish causal relationships between the factors assessed and burnout. Fourth, we used a non-proprietary, single-item measure of burnout that, while validated, may not be as comprehensive as the Maslach Burnout Inventory Human Services Survey for Medical Personnel [21]. Another limitation is the lack of information collected on different factors known to be related to burnout, such as organizational resources, work environment, depression or participants´ coping strategies [1, 22].

Identifying burnout may be critical for implementing preventive intervention strategies to maintain adequate functioning of memory clinics and neurology departments. Further studies with a longitudinal design and using validated comprehensive measurements are needed to understand the mechanisms involved in the impact of burnout on neurologists caring for patients with cognitive disorders.
